# Acute Pharmacologic Degradation of a Stable Antigen Enhances Its Direct Presentation on MHC Class I Molecules

**DOI:** 10.3389/fimmu.2017.01920

**Published:** 2018-01-08

**Authors:** Sarah C. Moser, Jane S. A. Voerman, Dennis L. Buckley, Georg E. Winter, Christopher Schliehe

**Affiliations:** ^1^Department of Immunology, Erasmus MC, University Medical Center Rotterdam, Rotterdam, Netherlands; ^2^Department for Medical Oncology, Dana-Farber Cancer Institute, Boston, MA, United States; ^3^CeMM Research Center for Molecular Medicine of the Austrian Academy of Sciences, Vienna, Austria

**Keywords:** MHC class I, antigen presentation, bifunctional degraders, PROTACs, protein degradation, DRiPs, immunotherapy, cancer, chronic infections

## Abstract

Bifunctional degraders, also referred to as proteolysis-targeting chimeras (PROTACs), are a recently developed class of small molecules. They were designed to specifically target endogenous proteins for ubiquitin/proteasome-dependent degradation and to thereby interfere with pathological mechanisms of diseases, including cancer. In this study, we hypothesized that this process of acute pharmacologic protein degradation might increase the direct MHC class I presentation of degraded targets. By studying this question, we contribute to an ongoing discussion about the origin of peptides feeding the MHC class I presentation pathway. Two scenarios have been postulated: peptides can either be derived from homeostatic turnover of mature proteins and/or from short-lived defective ribosomal products (DRiPs), but currently, it is still unclear to what ratio and efficiency both pathways contribute to the overall MHC class I presentation. We therefore generated the intrinsically stable model antigen GFP-S8L-F12 that was susceptible to acute pharmacologic degradation *via* the previously described degradation tag (dTAG) system. Using different murine cell lines, we show here that the bifunctional molecule dTAG-7 induced rapid proteasome-dependent degradation of GFP-S8L-F12 and simultaneously increased its direct presentation on MHC class I molecules. Using the same model in a doxycycline-inducible setting, we could further show that stable, mature antigen was the major source of peptides presented, thereby excluding a dominant role of DRiPs in our system. This study is, to our knowledge, the first to investigate targeted pharmacologic protein degradation in the context of antigen presentation and our data point toward future applications by strategically combining therapies using bifunctional degraders with their stimulating effect on direct MHC class I presentation.

## Introduction

Small molecules specifically targeting endogenous proteins for proteolytic degradation have been suggested as a novel therapeutic strategy against diseases, including cancer. While initially being limited to certain model systems depending on recombinant fusion proteins (1, 2) or rare examples of specific compound-induced degradation of endogenous proteins (3, 4), the development of bifunctional degraders, alias proteolysis-targeting chimeras, has opened new avenues to rationally target proteins for proteasomal degradation in a general, but specific fashion (5–9). Bifunctional degraders act by linking the protein of interest to a specific E3 ligase complex that in turn induces E2-mediated ubiquitination and proteasomal degradation of the recruited target. This strategy has recently been utilized to induce the degradation of BRD4, a transcriptional co-activator involved in cancer and other diseases (6–8, 10). Apart from their potent effect on protein stability, which consequently leads to the anticipated interference with the biological function of a given target, we hypothesized that bifunctional degraders might also alter the immunological visibility of degraded proteins by increasing their presentation on MHC class I molecules. This study therefore highlights a novel feature of therapies based on bifunctional degraders that could be used to increase CD8^+^ T-cell immunity towards cancer and intracellular infections.

Antigen presentation on MHC class I molecules enables. CD8^+^ cytotoxic T lymphocytes (CTLs) to screen the intracellular content of cells for signs of infection and/or transformation (11), ultimately leading to specific target cell destruction. In most cell types, the peptides for MHC class I presentation are exclusively derived from endogenously synthesized proteins that are degraded *via* the ubiquitin/proteasome system (12). A fraction of peptides escapes the complete recycling into amino acids and is subsequently transported to the endoplasmic reticulum *via* the transporter associated with antigen processing (13, 14). MHC class I loading of 8–10 amino acids long peptides can require additional trimming by specific aminopeptidases (15–17) and is supported by chaperones of the peptide-loading complex. Finally, loaded MHC molecules enter the secretory pathway *via* the Golgi apparatus to reach the cell surface. This pathway of MHC class I presentation is referred to as the “direct presentation pathway” and is distinct from MHC class I restricted cross-presentation of exogenously synthetized antigens by professional antigen presenting cells.

Our study of whether bifunctional degraders can enhance the direct MHC class I presentation of their targets, contributes to the ongoing characterization of the origin of peptides feeding the direct presentation pathway. Initially, these peptides were thought to be generated primarily during homeostatic turnover of mature proteins (retirees). However, this view could not explain the rapid MHC class I presentation of peptides derived from long-lived viral antigens. Therefore, an alternative hypothesis has been suggested that was based on the description of defective ribosomal products (DRiPs), short-lived polypeptides, and proteins (half-life <10 min) that are degraded before reaching their mature, functional stage (18). DRiPs are by definition derived from transcriptional and translational errors, premature termination of translation or protein misfolding and according to the DRiP-hypothesis they provide a highly efficient and rapid source of peptides for the direct presentation pathway (19, 20). Although being a field of intense research, it is still unclear to what ratio and efficiency DRiPs versus retirees contribute to the overall MHC class I presentation, and whether differences in the cellular state, including infections, immune signaling, levels of intracellular stress, or other cellular conditions can influence this ratio.

**Figure 1:**
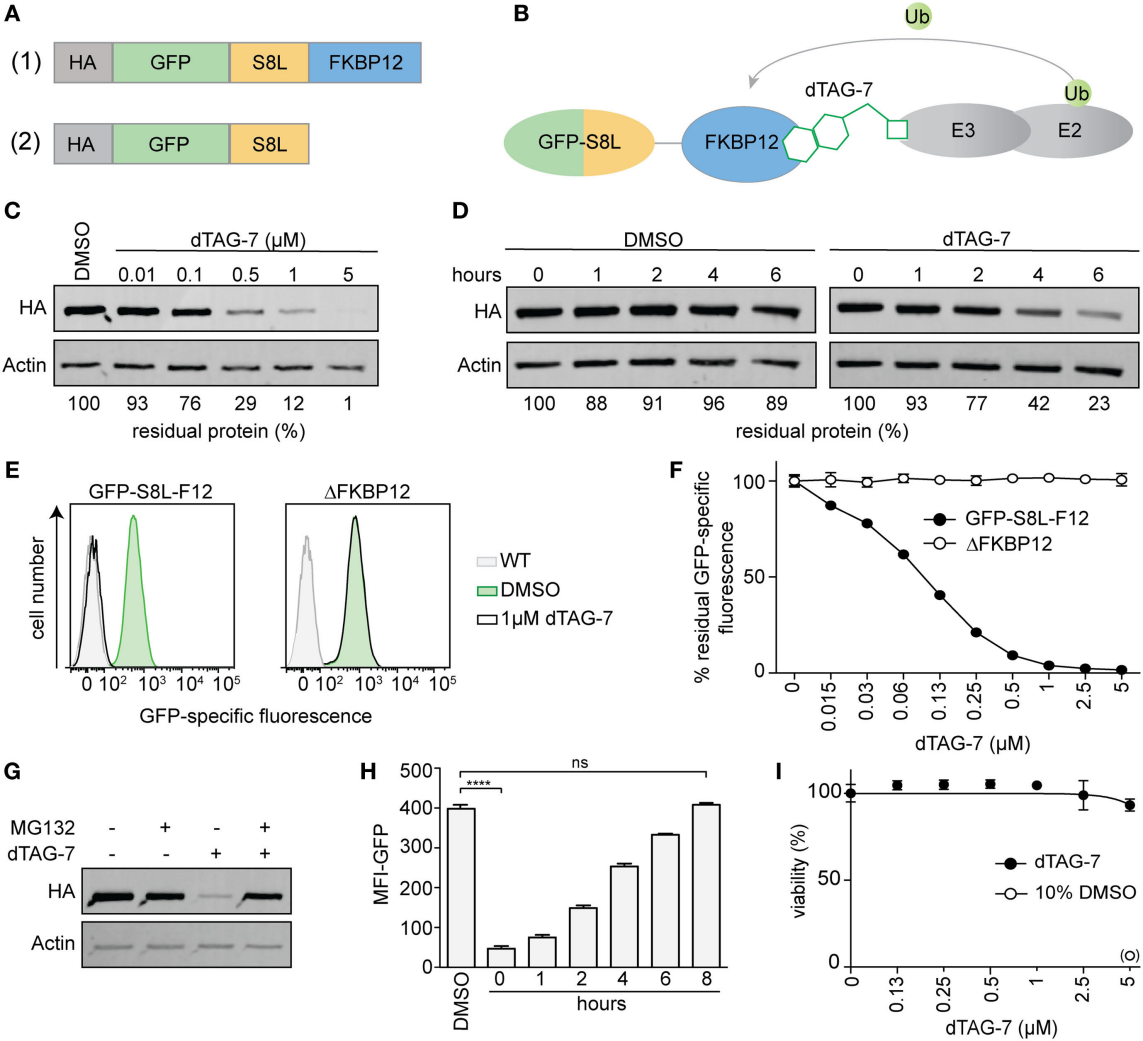
Generating a model system to study degradation tag (dTAG)-7-dependent antigen presentation. **(A)** Illustration of the model antigen GFP-S8L-F12 (1) and the control construct ΔFKBP12, lacking the dTAG (2). **(B)** Schematic illustration of the mechanism of dTAG-7-mediated antigen degradation. The dTAG-7 molecule shown does not reflect the original structure. **(C)** GFP-S8L-F12-expressing BMC-2 cells were treated with indicated concentrations of dTAG-7 or 0.1% DMSO as control and 18 h later the residual % of protein was assessed by anti-HA western blot analysis. Bands were quantified compared to the DMSO control and normalized to actin expression using ImageJ. **(D)** Antigen expressing BMC-2 cells were treated with 100 µg/mL of the translation inhibitor cycloheximide for indicated time points and 1 µM dTAG-7, or 0.1% DMSO as a control, was added to alter antigen stability. Residual amount of protein in % was analyzed by anti-HA western blot and quantified relative to actin expression using ImageJ. **(E,F)** Antigen- or ΔFKBP12-expressing BMC-2 cells were cultured in the presence of increasing concentrations of dTAG-7 or 0.1% DMSO as control (0 µM) for 18 h and GFP-specific fluorescence was quantified by flow cytometry. **(E)** Representative histograms comparing GFP-specific fluorescence with or without 1 µM dTAG-7 treatment to wild-type BMC-2 cells (WT). **(F)** The graph indicates % residual GFP-specific fluorescence normalized to the mean fluorescence intensity (MFI) measured with 0 µM dTAG-7, for each construct, respectively. Values are shown as the mean ± SD from triplicate measurements. **(G)** GFP-S8L-F12-expressing BMC-2 cells were cultured for 6 h in the presence or absence of 1 µM dTAG-7 and/or 10 µM of the proteasome inhibitor MG132. Afterward, GFP-S8L-F12 levels were evaluated by anti-HA western blot, using actin expression as control. **(H)** GFP-S8L-F12-expressing BMC-2 cells were treated with 1 µM dTAG-7 (0 h) or 0.1% DMSO as control for 18 h. Next, dTAG-7 was washed off and GFP-specific fluorescence (MFI-GFP) was analyzed by flow cytometry at indicated time points. Values are shown as the mean ± SEM from triplicate measurements. **(I)** ATP-based cell viability measurement (CellTiter-Glo) of GFP-S8L-F12-expressing BMC-2 cells treated for 18 h with indicated concentrations of dTAG-7 or 10% DMSO as a positive control for the induction of cell death. Values are shown as the mean ± SEM from triplicate measurements. Representative results from three **(C,D,G–I)** or two **(E,F)** independent experiments are shown.

In this study, we used dTAG-7, a previously characterized bifunctional degrader (9), to study the impact of acute pharmacologic antigen degradation on direct MHC class I presentation. In brief, dTAG-7 induces molecular proximity between the E3 ligase complex CRL4^CRBN^ and a target protein that is fused to a degradation tag (dTAG) derived from mutant FKBP12 (F36V). This proximity leads to selective and immediate proteasomal degradation of the target, as recently demonstrated for ENL, a factor important for leukemia pathogenesis (9). In analogy to previous reports using the stabilizing molecule Shield-1 that has opposite effects as compared to bifunctional degraders (21–24), we generated a model antigen fused to mutant FKBP12 which served as dTAG-7-dependent dTAG (Figures 1A,B). However, mechanistically two features brand this novel setup advantageous compared to previous experiments using Shield-1: first, fusion proteins of antigen and mutant FKBP12 in our system are intrinsically stable and do therefore more closely resemble the biology of cellular protein degradation, such as seen for retirees. Second, the addition of dTAG-7 leads to ubiquitin-dependent degradation *via* the ubiquitin E3 ligase complex CRL4^CRBN^ (Figure 1B) (7, 9), which in contrast to Shield-1-withdrawal, that primarily induces protein misfolding, is a considerably more defined process. Thus, the approach we have taken offers some significant advantages over the Shield-1 system.

We report here, that dTAG-7 induced rapid proteasome-dependent degradation of an intrinsically stable antigen-FKBP12 fusion protein and we observed increased MHC class I presentation of antigen-derived peptides upon dTAG-7 treatment on different murine cell lines. Using a doxycycline-inducible expression system we could further show that degradation of stable antigens provided the majority of peptides presented in our system. To our knowledge, this study is the first to investigate the strategy of acute pharmacologic target degradation in the context of antigen presentation. Not only do we provide further evidence that peptides derived from stable, mature antigens can serve as efficient source for the direct presentation pathway, but we also point out the potential of using bifunctional degraders to enhance direct MHC class I presentation of specific target proteins.

## Materials and Methods

### Cells and Media

The murine dendritic cell line DC2.4 (H-2^b^) (25) and the murine macrophage cell line BMC-2 (H-2^b^) (26) were originally obtained from K. Rock (University of Massachusetts Medical School, Worcester, MA, USA) and were cultured in RPMI 1640 (Lonza). The C57BL/6-derived methylcholanthrene-induced fibrosarcoma cell line MC57G (H-2^b^) (27) was grown in MEM (Gibco) and HEK293T (ATCC, CRL-3216) were cultured in DMEM (Lonza). All media were supplemented with 10% fetal calf serum (Gibco) and 100 U/mL penicillin/streptomycin (Lonza).

### Generation of Antigen Constructs

To generate the expression construct HA-GFP-S8L-FKBP12 (GFP-S8L-F12) (Figure 1A, top), we amplified eGFP from the vector pCMV-GFP (addgene #11153) using the primer pair 5′-TATGATGGTACCTATGGTGAGCAAGGGCGAG-3′ and 5′-ATCATAGGATCCCTTGTACAGCTCGTCCAT-3′ introducing a 5′-KpnI and a 3′-BamHI restriction site. The amplified fragment was then digested with KpnI and BamHI and ligated to an annealed double-stranded SIINFEKL (Ova_257–264_, S8L) oligo that additionally contained the 15 up- and downstream base pairs of the original sequence in ovalbumin, *via* a 3′-BamHI overhang. Next, GFP-S8L was ligated to annealed double-stranded HA oligos *via* the previously introduced KpnI restriction site at the 5′ end of GFP to generate the fragment HA-GFP-S8L. This fragment was again amplified by PCR using the primer pair 5′-CACCATGGCCTACCCCTACGAC-3′ and 5′-ATCATACTCGAGACTGGTCCATTCAGTCAG-3′ to generate a 3′-XhoI restriction site and a 5′-CACC-overhang required for Gateway cloning. Subsequently, FKBP12 (F36V) was amplified from pDONR-221-FKBP12^F36V^ (9) using the primer pair 5′-TATGATCTCGAGGGAGGCATGGGAGTGCAGGTGGAA-3′ and 5′-TCATTCCAGTTTTAGAAGCTCCACATC-3′ to introduce a 5′-XhoI restriction site. Finally, FKBP12 was ligated to HA-GFP-S8L *via* the 3′-XhoI site and cloned into the vector pENTR using the pENTR/D-TOPO-D-cloning kit (Invitrogen). The expression vectors based on pLex305 (constitutive, PGK promoter) and pCW57.1 (doxycycline-inducible) (addgene #41390, #41393) were generated using the Gateway LR clonase II enzyme mix (Invitrogen). The construct HA-GFP-S8L lacking the FKBP12 domain (ΔFKBP12) was amplified using the primer pair 5′-CACCATGGCCTACCCCTACGAC-3′ and 5′-TCAACTGGTCCATTCAGTCAGTT-3′ introducing a stop codon 3′ after the S8L sequence and the 5′CACC site for cloning into the vector pENTR and the expression vector pLex305 as described above.

### Lentivirus Production

HEK293T cells were seeded in 6-wells and transfected with 500 ng and 50 ng of the packaging plasmids psPAX2 and pCMV-VSV-G (Addgene #12260, #8454) and 500 ng of the expression vectors using Lipofectamine 2000 (Invitrogen). Supernatant was collected 24 and 48 h after transfection, pooled, sterile-filtered, and frozen at −80°C until use. Subsequently, cells were infected in full medium containing 8 µg/mL polybrene (Sigma) and centrifuged for 1.5 h at 30°C. 48 h after infection, cells were selected using 4 µg/mL puromycin (Sigma) for DC2.4 and BMC-2 cells and 6 µg/mL for MC57G cells. For all cell lines, single cell clones were generated by limiting dilution and analyzed for their GFP expression by flow cytometry.

### Western Blot Analysis

For testing degradation of the antigen, 4 × 10^5^ BMC-2 cells were seeded into 6-well plates, incubated overnight and treated with indicated concentrations of dTAG-7 [synthesized as described before (7)] or 0.1% DMSO as a control for 18 h. Then, samples were collected in cold PBS before further analysis. For western blot analysis, samples were lysed in RIPA buffer [50 mM Tris-HCl (pH = 8), 150 mM NaCl, 1% NP-40, 0.5% Na-deoxycholate, 0.1% SDS] supplemented with Halt Protease and Phosphatase Inhibitor Cocktail (Thermo Fisher) for 30 min on ice. After centrifugation, the supernatant was harvested and protein concentration was quantified using Bradford reagent (Sigma) and a VersaMax ELISA plate reader (Molecular Devices). Subsequently, samples were mixed with reducing sample buffer (80 mM Tris-HCl, 10% glycerol, 2% SDS, 5% β-mercaptoethanol, bromphenolblue), heated at 95°C for 10 min, loaded onto SDS-PAGE Gels (4–15% Mini-Protean TGX Gels, Bio-Rad) and run at 120 V. Then samples were transferred onto a PVDF-membrane (Millipore) using a Bio-Rad blotting system, and membrane was blocked in 5% milk powder in PBS containing 0.05% Tween-20 (PBS-T) for 1 h. Membranes were stained with a mouse anti-HA (clone HA-7, Sigma) and a polyclonal rabbit anti-β-actin antibody (Sigma) in 2.5% milk powder in PBS-T overnight, washed with PBS-T, incubated with goat anti-mouse IRDye800CW and goat anti-rabbit IRDye680-labeled secondary antibodies (Li-Cor) for 1 h, and imaged with an Odyssey Infrared Imager (Li-Cor). Quantification of the blots was performed using ImageJ version 1.5.1. Uncropped images of western blots are displayed in Figure S1 in Supplementary Material.

### Cycloheximide Chase

In order to assess antigen stability, 4 × 10^5^ BMC-2 cells were seeded in 6-well plates, incubated overnight and then treated with 100 µg/mL of the translation inhibitor cycloheximide (Sigma), 1 µM dTAG-7 or 0.1% DMSO as a control. Samples were collected in cold PBS at indicated time points and protein levels were analyzed by western blot.

### Proteasome Inhibition

To investigate proteasome dependency of dTAG-7-mediated degradation, 4 × 10^5^ BMC-2 cells were seeded in 6-well plates, incubated overnight, and treated for 6 h in the presence or absence of 1 µM dTAG-7 and/or 10 µM of the proteasome inhibitor MG132 (Merck Chemicals) as indicated. Subsequently, cells were collected in cold PBS and protein levels were measured by western blot analysis.

### Cell Viability Assay

To determine the toxicity of dTAG-7, 5 × 10^3^ BMC-2 cells were seeded in a 96-well plate and incubated with indicated concentrations of dTAG-7 (1 mM stock in DMSO) for 24 h or 10% DMSO as positive control for induction of cell death. Subsequently, the supernatant was removed by centrifugation and cells were resuspended in Cell Titer Glo reagent (Promega), according to the manufactures protocol. Luminescence was quantified using a GloMax Explorer (Promega), and values were normalized to a control treated with 0.1% DMSO, representing the highest concentration of dTAG-7.

### Quantification of MHC Class I Presentation

To quantify H-2K^b^/S8L presentation, 2 × 10^5^ cells were treated as indicated, resuspended in 50 µL FACS buffer (0.1% NaN_3_, 0.5% BSA in PBS) containing a monoclonal anti-H-2K^b^/S8L antibody, which specifically recognizes S8L (OVA_257–264_) bound to the MHC class I molecule H-2K^b^ (25D1.16, APC-labeled, 1:160, eBioscience) and incubated on ice for 30 minutes. Cells were washed twice with 150 µL FACS buffer. Then, samples were resuspended in 200 µL FACS buffer and measured on a FACS Canto II (BD Biosciences). GFP-specific fluorescence was measured in parallel. Total MHC class I presentation was detected using anti-H-2K^b^ and anti-H-2D^b^ antibodies (1:200, PE-labeled, E-Bioscience), and the staining was performed for 20 min at 4°C. Using the FlowJo software (Version 7.6 (Treestar)), samples were gated on all living cells *via* forward and side scatter and background mean fluorescence intensities (MFI) (non-transduced or non-doxycycline treated cells, respectively, stained with the 25D1.16 antibody) were subtracted from all values. The impact of dTAG-7-mediated antigen degradation on GFP-specific fluorescence and H-2K^b^/S8L presentation was tested by seeding 2 × 10^5^ cells in triplicates in 96-well plates. 1 µM dTAG-7 was added 30, 24, 18, 8, 6, 4, 2, 1.5, 1, and 0.5 h before collection to induce degradation of the fusion antigen. To exclude any influence of DMSO alone, cells were incubated with 0.1% DMSO, corresponding to the amount of DMSO in samples treated with 1 µM dTAG-7, for 30 h. After treatment with dTAG-7, cells were centrifuged and stained for flow cytometry as described above. To investigate the impact of newly synthesized antigen on H-2K^b^/S8L presentation, 2 × 10^5^ cells were seeded in 96-well plates, treated with 1 µg/mL doxycycline (Sigma) and indicated concentrations of dTAG-7 for 3 and 6 h. Then cells were stained as mentioned above. The contribution of stable antigens to the pool of MHC class I substrates was calculated as % dTAG-7 dependent H-2K^b^/S8L presentation = (1 − ((H-2K^b^/S8L)_no dTAG-7_/(H-2K^b^/S8L)_+1µM dTAG-7_)) × 100.

### Real-time PCR

4 × 10^5^ BMC-2 cells were treated with 1 µg/mL doxycycline to induce antigen expression. After 24 h, cells were washed twice with PBS, cultured again in full medium, and collected in QIAzol Lysis Reagent (Qiagen) at indicated time points. RNA was isolated using the recommended phenol-chloroform extraction protocol and quantified using a NanoDrop One Microvolume UV-Vis spectrophotometer (VWR). 1 µg of RNA was transcribed to cDNA using the RevertAid First Strand cDNA Kit (Thermo Fisher). Real-time PCR was performed using TaqMan Universal PCR Master Mix (Thermo Fisher) and the following primers: GFP: 5′-CTGCTGCCCGACAACCAC-3′, 5′-TGTGATCGCGCTTCTCGTT-3′, probe: 5′-FAM-ACCTGAGCACCCAGTCCGCCCT-TAMRA-3′; Eef1a1: 5′-GCAAAAACGACCCACCAATG-3′, 5′-GGCCTGGATGGTTCAGGATA-3′, probe: 5′-FAM-CACCTGAGCAGTGAAGCCAG-TAMRA-3′. The assay was run on a Quant Studio 5 Real-Time PCR System (Applied Biosystems) in triplicates and expression was normalized to the housekeeping gene Eef1a1 (eukaryotic translation elongation factor 1 alpha 1). To quantify the expression of inflammatory genes in cells treated with toll-like receptor (TLR) stimulants, 4 × 10^5^ cells were seeded in triplicates in 6-well plates. Treatment was performed by adding 20 ng/mL lipopolysaccharide (LPS) or 6 µg/mL poly(I:C) (both Invivogen) the next day. Samples were collected after 8 h in QIAzol Lysis Reagent (Qiagen), RNA was isolated and real-time PCR performed as mentioned above, using the TaqMan gene expression assays *IL-6* (Mm00446190) and *Mx1* (Mm00487796) (Thermo Fisher).

### Statistics

All results are shown as the mean ± SEM from experimental triplicates, unless indicated otherwise. Significance was calculated by student’s *t*-tests using GraphPad Prism 5 (ns, not significant; *p ≤ 0.05, **p ≤ 0.01, ***p ≤ 0.001, ****p ≤ 0.0001).

## Results

### Generating a Model System to Study dTAG-7-Dependent Antigen Presentation

In order to investigate the impact of dTAG-7-mediated antigen degradation on its MHC class I presentation, we generated a model system that would allow us (a) to monitor antigen expression and presentation and (b) to control antigen degradation using the small molecule dTAG-7. Therefore, we fused the well-studied H-2K^b^-restricted T-cell epitope SIINFEKL (S8L) derived from chicken ovalbumin to the C-terminus of enhanced GFP that served as fluorescent reporter for flow cytometry. In addition, we added a mutant version of FKBP12 (F36V) to the C-terminus of GFP-S8L to act as a dTAG-7-dependent dTAG (7, 9) (Figure 1A, top, Figure 1B). Finally, an N-terminal hemagglutinin (HA) tag was added to allow detection by western blot. This construct was transduced into the C57BL/6-derived macrophage cell line BMC-2 (H-2^b^) using a lentiviral delivery system to generate stable clones with constitutive expression. To demonstrate dTAG-7-dependent degradation of HA-GFP-S8L-FKBP12 (from here on called “GFP-S8L-F12”) in BMC-2 cells, we treated cells with increasing concentrations of dTAG-7 and measured GFP-S8L-F12 expression by western blot (Figure 1C). dTAG-7 triggered the degradation of GFP-S8L-F12 in a concentration-dependent manner, with minimal residual protein detected at 5 µM. A similar range of dTAG-7 activity has previously been observed in human cell lines (9) and importantly, dTAG-7 treatment had no influence on the expression of actin that served as control. Next, we performed a cycloheximide chase to demonstrate the decrease in antigen stability after dTAG-7 treatment (Figure 1D). From this experiment, we could conclude that GFP-S8L-F12 was intrinsically stable (~90% after 6 h) and that addition of dTAG-7 led to a reduction of its half-life (~20% after 6 h). Since western blot analysis is a measure of total protein (properly folded as well as misfolded), we further employed GFP-specific fluorescence as readout for the degradation of properly folded, mature GFP-S8L-F12 (Figures 1E,F). dTAG-7 triggered a reduction of the GFP-specific fluorescence in a concentration dependent manner, and samples treated with 1 µM dTAG-7 already reached values close to baseline. To further demonstrate the target specificity of dTAG-7, we additionally generated stable BMC-2 cells expressing a construct lacking the FKBP12 domain of GFP-S8L-F12 (ΔFKBP12) (Figure 1A, bottom). GFP-specific fluorescence of cells expressing ΔFKBP12 was not altered by increasing concentrations of dTAG-7, indicating that GFP-S8L-F12 degradation in this system is indeed FKBP12 dependent (Figures 1E,F). To further characterize our system, we aimed to confirm dTAG-7-specific degradation to be proteasome dependent. Therefore, we induced GFP-S8L-F12 degradation in the presence or absence of the proteasome inhibitor MG132 (Figure 1G). While treatment of cells with dTAG-7 alone lead to degradation of GFP-S8L-F12, inhibition of the proteasome reversed this effect, indicating that GFP-S8L-F12 degradation in this system was indeed proteasome dependent. To demonstrate that dTAG-7 treatment did not irreversibly affect GFP-S8L-F12 expression or the degradation machinery, we performed a wash-out experiment, in which cells were pre-treated with dTAG-7 for 18 h before the compound was removed and GFP-specific fluorescence was followed over time (Figure 1H). This experiment indicated that dTAG-7-induced degradation is completely reversible and already 8 h after wash-out of the compound GFP-S8L-F12 expression is restored to pretreatment levels. In line with this, dTAG-7 did not show cytotoxicity at the concentrations used in this study (Figure 1I). Together, these experiments demonstrate that we generated an expression system that could be used to study the impact of dTAG-7-mediated antigen degradation on its MHC class I presentation.

### dTAG-7-Induced Antigen Degradation Increases Its MHC Class I Presentation

**Figure 2:**
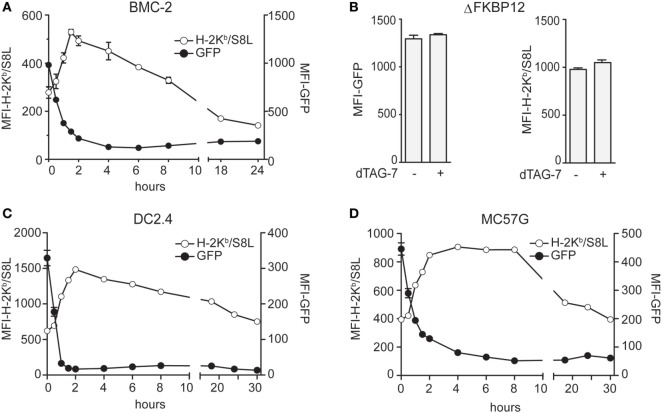
Degradation tag (dTAG)-7-induced antigen degradation increases its MHC class I presentation. **(A)** GFP-S8L-F12-expressing BMC-2 cells were treated with 1 µM dTAG-7 for indicated times. GFP-specific fluorescence (MFI-GFP) and MHC class I presentation of antigen-derived S8L (MFI-H-2K^b^/S8L) was detected by flow cytometry. MFI, mean fluorescence intensity. **(B)** BMC-2 cells expressing ΔFKBP12 were treated for 2 h with 1 µM dTAG-7 or left untreated as control and MFI-GFP as well as MFI-H-2K^b^/S8L were measured by flow cytometry as described above. **(C,D)** GFP-S8L-F12-expressing DC2.4 **(C)** and MC57G **(D)** cells were treated and analyzed as described in **(A)**. All experiments were performed in triplicates and results are shown as mean ± SEM. Representative results from three independent experiments are shown.

After having established the experimental setup, we next set out to test the impact of dTAG-7-mediated GFP-S8L-F12 degradation on its direct MHC class I presentation. We therefore treated GFP-S8L-F12-expressing BMC-2 cells with dTAG-7 and followed GFP-specific fluorescence, as well as S8L presentation using an H-2K^b^/S8L-specific antibody, over time by flow cytometry (Figure 2A). In line with the experiments shown above, we could observe a strong decrease in GFP-specific fluorescence already during the first hours after dTAG-7 treatment. At the same time, a prominent increase in H-2K^b^/S8L surface exposure could be detected with a peak at around 2 h after dTAG-7 treatment. To exclude any non-specific effects on the MHC class I presentation machinery, we performed the same experiment using cells expressing ΔFKBP12 (Figure 2B). Here, GFP-specific fluorescence as well as presentation of H-2K^b^/S8L was comparable before and 2 h after dTAG-7 treatment, the time when maximal increase in H-2K^b^/S8L presentation was observed using complete GFP-S8L-F12. In addition, we treated wild-type BMC-2 cells with dTAG-7 and measured the overall MHC class I surface expression (Figure S2 in Supplementary Material, left part). As expected, we did not observe any alterations in total H-2K^b^ and H-2D^b^ presentation after dTAG-7 treatment. These experiments indicated that the dTAG-7-dependent effect on MHC class I presentation was antigen-specific and FKBP12-dependent. Further, we also measured total MHC class I presentation of GFP-S8L-F12-expressing BMC-2 cells. While dTAG-7 treatment did not alter the total surface expression of H-2D^b^, we observed a significant increase in the overall H-2K^b^ presentation on these cells (Figure S2 in Supplementary Material, right part). This demonstrates that epitope generation seems to be rate-limiting for MHC class I presentation under steady-state conditions and that the amount of H-2K^b^/S8L presentation after dTAG-7-mediated degradation of GFP-S8L-F12 is strong enough to increase the total levels of H-2K^b^. Next, we aimed to further confirm these findings in GFP-S8L-F12-expressing DC2.4 cells of dendritic cell origin and in the fibrosarcoma cell line MC57G (both H-2^b^). In both, DC2.4 cells (Figure 2C) and MC57G cells (Figure 2D) addition of dTAG-7 induced a strong reduction of GFP-specific fluorescence within the first 2 h of treatment, indicating efficient dTAG-7-specific GFP-S8L-F12 degradation. In agreement with the results seen with BMC-2 cells, the simultaneous measurement of GFP-S8L-F12-derived surface expression of H-2K^b^/S8L revealed that the dTAG-7-induced antigen degradation in DC2.4 and MC57G cells strongly increased the MHC class I presentation of S8L. Overall, we concluded from these experiments that dTAG-7-mediated antigen degradation enhanced MHC class I presentation of its target.

### Stable Antigen Is the Major Source of MHC Class I Presentation upon dTAG-7 Treatment

**Figure 3:**
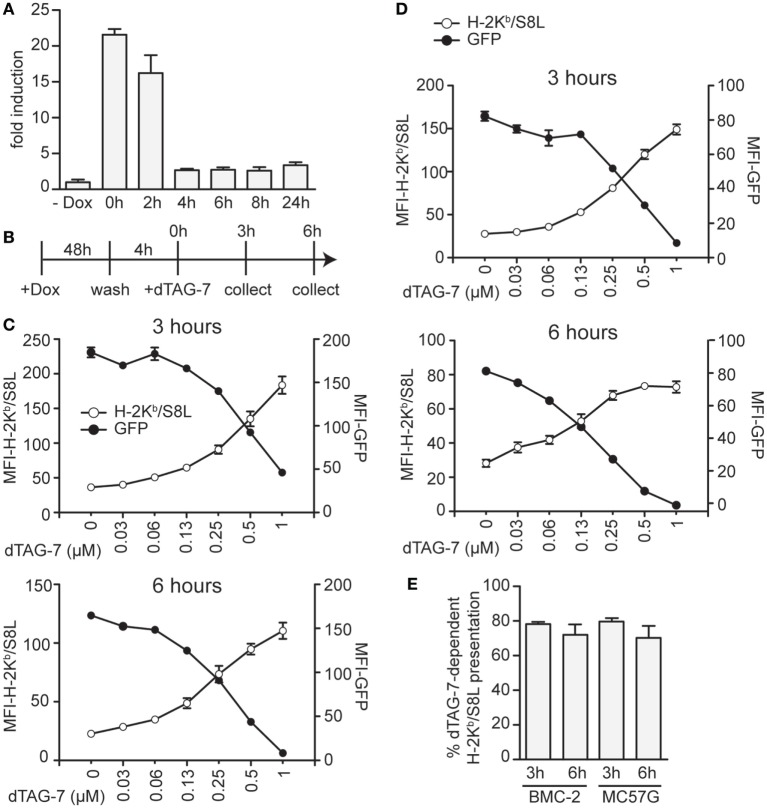
Stable antigen is the major source of MHC class I presentation upon degradation tag (dTAG)-7 treatment. **(A)** Real-time PCR analysis to measure the stability of *GFP-S8L-F12* mRNA. BMC-2 cells were treated with 1 µg/mL doxycycline to induce GFP-S8L-F12 expression for 24 h. Then, doxycycline was washed off and mRNA stability was followed for indicated times. *GFP-S8L-F12* expression is shown as fold induction compared to basal levels in the absence of doxycycline (- Dox). **(B)** Schematic illustration of the experiments performed in **(C,D)**. **(C,D)** GFP-S8L-F12 expression in BMC-2 **(C)** and MC57G cells **(D)** was induced by doxycycline for 48 h. Next, doxycycline was removed for 4 h, before cells were treated with 1 µM dTAG-7 for 3 or 6 h, respectively. Afterward, GFP-specific fluorescence (MFI-GFP) and MHC class I presentation of S8L (MFI-H-2K^b^/S8L) were quantified by flow cytometry. MFI, mean fluorescence intensity. **(E)** Quantification of the results shown in **(C,D)** from three independent experiments (for calculation details see Materials and Methods). All experiments were performed in triplicates and results are shown as mean ± SEM. Representative results from three independent experiments are shown.

We next aimed to test the impact of dTAG-7-mediated antigen degradation in the absence of DRiPs and therefore chose to perform additional experiments in a doxycycline-inducible setting. Using the same lentiviral strategy, we generated stable cell lines expressing our model antigen GFP-S8L-F12 in a doxycycline-dependent manner. As DRiPs by definition have a very short half-life (<10 min) (18), doxycycline removal from GFP-S8L-F12-expressing cells should, therefore, depending on the residual stability of *GFP-S8L-F12* mRNA, strongly reduce their presence. As a consequence, the previously accumulated pool of stable antigen would remain the only source to fuel the MHC class I presentation pathway. We, therefore, first induced GFP-S8L-F12 expression in BMC-2 cells to generate a pool of stable antigen and then removed doxycycline to stop its transcription. In order to determine the stability of *GFP-S8L-F12* mRNA, we took samples at indicated time points after doxycycline withdrawal and performed a quantitative real-time PCR analysis (Figure 3A). According to these data, already 4 h after doxycycline withdrawal the amount of GFP-S8L-F12 mRNA was strongly reduced. Next, we induced expression of GFP-S8L-F12 in BMC-2 cells for 48 h, removed doxycycline for 4 h to reduce DRiP production, and then induced antigen degradation by dTAG-7 for 3 and 6 h (Figure 3B). Similar to the results observed before, dTAG-7-mediated antigen degradation was associated with a strong increase in H-2K^b^/S8L presentation at both time points measured (Figure 3C). As before, these results could also be confirmed when using doxycycline-inducible MC57G cells as an independent validation (Figure 3D). We next quantified the percentage of H-2K^b^/S8L presentation that was derived from dTAG-7-mediated GFP-S8L-F12 degradation. This quantification revealed that after DRiP-depletion, dTAG-7-mediated GFP-S8L-F12 degradation contributed around 70–80% to the overall H-2K^b^/S8L presentation (Figure 3E). These results demonstrate the importance of stable antigens in serving as a substrate for MHC class I presentation in this system.

### Minor Contribution of DRiPs to the Direct MHC Class I Presentation of GFP-S8L-F12-Derived S8L

**Figure 4:**
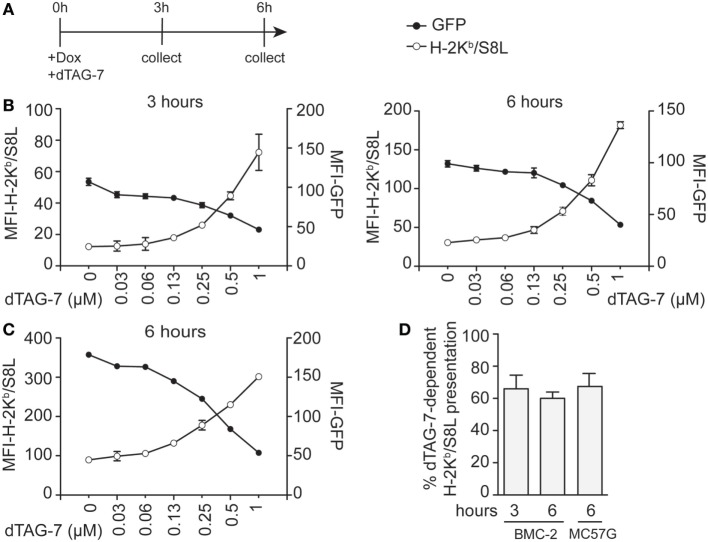
Minor contribution of defective ribosomal products to the direct MHC class I presentation of GFP-S8L-F12-derived S8L. **(A)** Timeline of the experiments performed in **(B,C)**. **(B,C)** BMC-2 cells **(B)** or MC57G cells **(C)** were simultaneously treated with 1 µg/mL doxycycline (Dox) and 1 µM degradation tag (dTAG)-7 to induce GFP-S8L-F12 expression and degradation at the same time. Next, GFP-specific fluorescence (MFI-GFP) and MHC class I presentation of GFP-S8L-F12-derived S8L (MFI-H-2K^b^/S8L) was detected by flow cytometry after 3 and 6 h, as indicated. MFI, mean fluorescence intensity. **(D)** Quantification of three independent experiments performed in **(B,C)** (for calculation details see Materials and Methods). All experiments were performed in triplicates and results are shown as mean ± SEM. **(B,C)** Representative results from three independent experiments are shown.

The previously observed increase in MHC class I-restricted GFP-S8L-F12 presentation upon dTAG-7 treatment originated from rapid degradation of a pool of stable antigen that accumulated in the cells over time. In order to further estimate the potential of DRiPs in our system, we next chose a setting that allowed DRiPs and mature antigens to contribute equally to the overall MHC class I presentation with regard of the time-of-synthesis ratio (23). We induced GFP-S8L-F12 expression in BMC-2 cells by addition of doxycycline and simultaneously started antigen degradation by adding increasing concentrations of dTAG-7 (Figure 4A). In this setting, it is important to highlight that degraded dTAG-7 substrates are by definition not converted into DRiPs, as they first need to acquire a mature conformation in order to being targeted. We quantified GFP-specific fluorescence and surface presentation of H-2K^b^/S8L after 3 and 6 h (Figure 4B). Similar to the experiments with constitutive GFP-S8L-F12 expression and DRiP-depletion, we observed a strong dTAG-7-dependent increase in H-2K^b^/S8L presentation, indicating that also without previous generation of an antigen pool, degradation of the mature, stable antigen contributed a major part to the overall MHC class I presentation of this antigen. These results could also be confirmed when using doxycycline-inducible MC57G cells (Figure 4C). To quantify the amount of H-2K^b^/S8L presentation derived from dTAG-7-mediated antigen degradation, we again calculated the percentage of dTAG-7-dependent H-2K^b^/S8L presentation, as above (Figure 4D). Interestingly, using this experimental setting, we consistently observed slightly lower percentages for the contribution of dTAG-7-mediated degradation of the mature, stable GFP-S8L-F12 of around 60–70% of the overall presented peptides. This number was around 10% lower than compared to the previous quantification after DRiPs depletion and we conclude from these experiments that this difference in H-2K^b^/S8L presentation might reflect the contribution of DRiPs in our system.

### dTAG-7-Mediated Antigen Presentation Is Not Altered by Immune Stimulation

**Figure 5:**
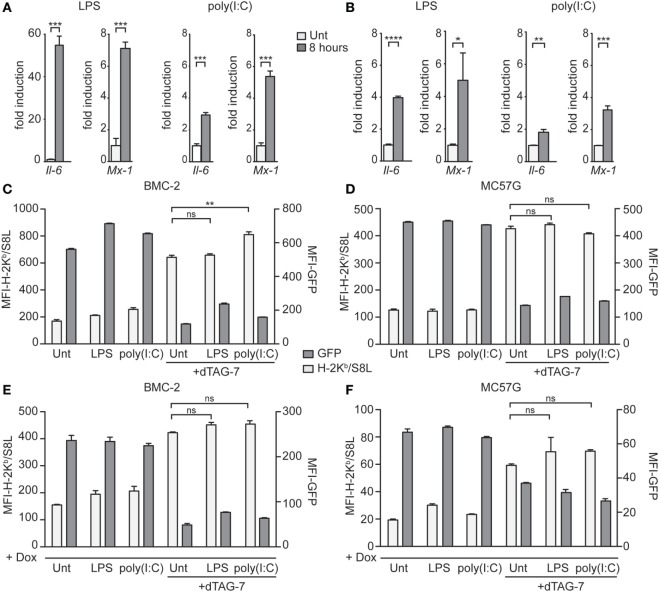
Degradation tag (dTAG)-7-mediated antigen presentation is not altered by immune stimulation. **(A,B)** Real-time PCR analysis of the inflammatory genes *Il-6* and *Mx-1*, 8 h after treatment of BMC-2 **(A)** and MC57G cells **(B)** with either 20 ng/mL lipopolysaccharide (LPS) or 6 µg/mL poly(I:C). Results are shown as fold induction compared to the untreated control (Unt). Gene expression was normalized to the housekeeping gene *Eef1a1*. **(C,D)** GFP-S8L-F12-expressing BMC-2 cells **(C)** or MC57G **(D)** were treated with LPS and poly(I:C) for 24 h to induce the expression of inflammatory genes. Next, cells were incubated in the presence or absence of 1 µM dTAG-7 for 2 h, before GFP-specific fluorescence (MFI-GFP) and presentation of GFP-S8L-F12-derived S8L (MFI-H-2K^b^/S8L) was detected by flow cytometry. MFI, mean fluorescence intensity. **(E,F)** BMC-2 **(E)** and MC57G **(F)** cells were simultaneously treated with toll-like receptor agonists and 1 µg/mL doxycycline (Dox) in the presence or absence of 1 µM dTAG-7. After 6 h, MFI-GFP and MFI-H-2K^b^/S8L were detected as described above. All experiments were performed in triplicates and results are shown as mean ± SEM. Representative results from three independent experiments are shown.

Therapies based on bifunctional degraders aim to interfere with critical mechanisms of diseases, including cancer. Often, such mechanisms develop in a microenvironment with pronounced inflammation and immune cell activation. To test whether the enhancing effect of dTAG-7 treatment on MHC class I presentation can also be seen under inflammatory conditions, we treated GFP-S8L-F12-expressing cells with different TLR agonists before inducing dTAG-7-mediated antigen degradation. Since the contribution of DRiPs to direct MHC class I presentation has been thought to be of special importance during cellular infections (18, 28), these experiments also tested the role of DRiPs under inflammatory conditions in our model. First, we investigated whether our antigen-expressing cell lines could be induced by TLR stimulation. Therefore, we measured the expression of inflammatory genes in BMC-2 and MC57G cells in response to the TLR4 agonist LPS, and the TLR3 agonist and viral RNA mimic poly(I:C) (Figures 5A,B). Indeed, we could show that TLR-stimulated cells significantly induced the expression of the NFκB target gene *Il-6*, as well as the interferon-stimulated gene *Mx1*. We next pretreated BMC-2 cells with TLR agonists for 24 h to induce the expression of inflammatory genes and then cultured cells in the presence or absence of dTAG-7 (Figure 5C). Similar to the experiments performed before, we could observe pronounced dTAG-7-mediated GFP-S8L-F12 degradation as measured by reduction in GFP-specific fluorescence. Interestingly, TLR stimulation did not reduce the potency of dTAG-7 to increase H-2K^b^/S8L presentation, as expected if a DRiP-associated pathway would show a more dominant role under these conditions. In fact, TLR stimulation rather increased H-2K^b^/S8L presentation after dTAG-7 treatment, probably due to general upregulation of the MHC class I presentation machinery. The same results could also be observed using antigen-expressing MC57G cells (Figure 5D). To mimic the role of DRiPs shortly after infection, we performed experiments in which TLR stimulation and induction of GFP-S8L-F12 expression were performed simultaneously. Also in this setting, for BMC-2 cells (Figure 5E), as well as for MC57G cells (Figure 5F), we did not observe a reduction in dTAG-7-induced H-2K^b^/S8L presentation 6 h after TLR stimulation. From these experiments, we conclude that the observed effect of dTAG-7 in boosting MHC class I presentation of its target works independently of the inflammatory status of the target cell that we induced in this study. At the same time, our results demonstrate that stimulation of antigen-expressing cells with LPS or poly(I:C) did not increase the contribution of DRiPs to the H-2K^b^/S8L presentation.

## Discussion

The therapeutic impact of direct pharmacologic protein degradation has thus far only been assessed by addressing the direct disease-specific addictions to the target of interest (5–10). In our study, we hypothesized that protein degradation using bifunctional molecules might in addition affect the direct MHC class I presentation of degraded targets. We generated cell lines expressing the model antigen GFP-S8L-F12 that was found to be intrinsically stable (~90% after 6 h) and susceptible for dTAG-7-mediated proteasome-dependent degradation. Using this model system, we could show in various experimental setups and murine cell types, including a fibrosarcoma, a dendritic, and a macrophage cell line, that the induction of antigen degradation by dTAG-7 strongly increased the direct MHC class I presentation of the degraded target.

This study adds to previous discussions about the origin of peptides feeding the direct MHC class I presentation pathway, a question that has been studied intensively over the last decades and that is still controversial in the literature (20, 29–31). The original idea that homeostatic turnover of mature proteins, so called retirees, serves as primary source for antigenic peptides has been challenged by the DRiP-hypothesis, which until recently has become a well-accepted model in the field (19). However, with novel technologies emerging, the DRiP-hypothesis is being re-evaluated and the initial model of homeostatic turnover of retirees is experiencing renewed attention.

It has long been difficult to manipulate the stability of model antigens without altering transcription and/or translation in general. Therefore, previous studies estimating the role of DRiPs versus retirees used, e.g., general translation inhibitors such as cycloheximide (23, 32–34) or ubiquitin fusion proteins (35, 36) to inhibit DRiP production or to alter the stability of model antigens. This changed with the development of the cell-permeable small molecule compound Shield-1 that reversibly stabilizes a rapidly degraded mutant version of the protein FKBP12 (21). As a consequence, fusion proteins of mutant FKBP12 and model antigens were shown to be unstable and rapidly degraded by the proteasome, unless being stabilized by Shield-1 (21, 23, 24). This system, for the first time, provided an experimental setup to study the impact of antigen stability on MHC class I presentation without altering transcription or translation. However, two recent studies using the Shield-1 system came to different conclusions when aiming to elucidate the role of DRiPs versus retirees in MHC class I presentation (23, 24). While Dolan et al. saw little effect of Shield-1-stabilized antigen on the total amount of antigen-specific MHC class I presentation and interpreted this as evidence for a major contribution of DRiPs (23), the study by Colbert et al. found a dominant role of stable/mature antigens in feeding the MHC class I presentation pathway, interestingly using similar antigen constructs and cellular systems (24).

Bifunctional degraders like dTAG-7 have the opposite effect of stabilizers such as Shield-1 (6–9, 21), but similarly they can be used to manipulate antigen stability. To allow direct comparison with the existing literature using Shield-1, we chose to study dTAG-7-mediated degradation using a model antigen that we generated in analogy to previous studies (23, 24). In agreement with the report by Colbert et al. (24), our study showed that dTAG-7-mediated degradation of a mature antigen increased its MHC class I presentation. Using a doxycycline inducible system we further analyzed the contribution of DRiPs in our settings. After antigen induction, doxycycline withdrawal led to a rapid depletion of antigen mRNA and allowed us to study antigen presentation in a context that by definition should be devoid of DRiPs, which are extremely short-lived and require neosynthesis. Here, dTAG-7-mediated antigen degradation contributed around 80% to the overall MHC class I presentation. It was previously discussed in the literature that degradation of a large pool of antigen in a short period of time might lead to an underestimation of the importance of DRiPs (23, 24). Therefore, we induced antigen expression and degradation simultaneously, generating a situation in which no previous antigen pool could be formed. Here, DRiPs and dTAG-7-mediated antigen degradation should have the same time-of-synthesis contribution (23) to the overall MHC class I presentation and the observed effect of dTAG-7 should therefore be interpreted to occur in direct competition with DRiPs. Interestingly, also in this experimental setting, degradation of stable antigen was found to be the major contributor to the overall MHC class I presentation. However, the calculated ratio for the contribution of stable antigen in this setting was around 10% lower as compared to the experiments with doxycycline withdrawal. We interpret presentation in the absence of dTAG-7 as the sum of DRiPs and first-order degradation independent of dTAG-7 treatment (30). Although it is difficult to further dissect this residual presentation capacity and we cannot entirely exclude the persistence of DRiP-derived H-2K^b^/S8L presentation even 10 h after doxycycline withdrawal, we suggest that the 10% difference between the two settings using doxycycline-inducible expression can be attributed to DRiPs. The remaining 20% seen after doxycycline withdrawal are likely contributed by first-order degradation, which is in agreement with the detected levels of dTAG-7-independent degradation (Figure 1D). GFP-based antigens, such as GFP-S8L-F12, might in general have a higher DRiP-rate compared to other antigens due to a process of auto-fragmentation (37). Therefore, the dominant role of stable antigens that we observed in our system could be even more pronounced for physiologic antigens.

Defective ribosomal products were proposed to be important during infections (28, 38, 39), and it was previously suggested that immune stimulation could potentially promote the generation and utilization of DRiPs (29, 40). Therefore, we hypothesized that this could be a possible explanation for differences reported in the Shield-1 system. While Colbert et al. used a sterile trigger to induce antigen expression (doxycycline), Dolan et al. employed a recombinant vaccinia virus to deliver the antigen (23, 24). In this direct comparison, viral delivery could have activated cellular immune signaling pathways potentially increasing the influence of DRiPs in this system. To look into this possibility, we studied the impact of dTAG-7-mediated antigen degradation in the presence of inflammatory stimuli. Interestingly, the dTAG-7-induced increase in MHC class I presentation was observed regardless of whether naïve or TLR-stimulated cells were analyzed. These results indicate that, at least in the cell lines and with the stimulations used in this study, the relative contribution of DRiPs versus dTAG-7-targeted antigens did not depend on the inflammatory status of the cells. With regard to the strategy of using bifunctional degraders for the treatment of diseases, these results indicate that the stimulating effects we described for MHC class I presentation could also be applied under inflammatory conditions, such as seen in cancer or during infections.

Our study uncovered a novel characteristic of bifunctional degraders that suggests additional modes of action for related therapies. Extrapolating from our study, we suggest that the enhancing effect on MHC class I presentation could be beneficial for several treatment scenarios: bifunctional degraders might increase the immunological visibility of treated cells toward pre-existing CTL responses in cancer and acute or chronic infections. Also, bifunctional molecules with different target-specificities could be combined to degrade (a) key players of disease progression and (b) e.g., prominent (neo-) antigens in cancer or infectious disease. Further, the stimulating effects on MHC class I presentation could be combined with checkpoint inhibitor treatment (41). Finally, bispecific molecules could also be combined with CTL-vaccines of the same antigen specificity to further boost class I-restricted responses against specific targets (42).

However, our experiments also highlight a limitation for immunotherapies using degraders, as the increasing effect on direct MHC class I presentation is transient. The strong induction observed after dTAG-7 treatment slowly returned to baseline levels, in line with the fact that ongoing degradation depletes the pool of mature antigen. Therefore, in an immunotherapeutic setting, phases of high compound activity, corresponding to target degradation with increased MHC class I presentation, could be alternated with phases of low activity, thereby recharging the antigen pool. Depending on the metabolic stability of bifunctional molecules *in vivo*, it will therefore be important to calculate the effective compound activity in a specific tissue of interest and to aim for an oscillating concentration with regular phases of suboptimal levels leading to re-accumulation of the antigen.

Our study has revealed a novel immune-stimulating property of bifunctional degraders. Further research will be needed to fully estimate their potential in promoting MHC class I presentation of specific targets in the context of diseases. Also, we provided further evidence that stable/mature antigen can serve as a prominent source of peptides feeding the MHC class I presentation pathway. We anticipate that our study will trigger further investigations making use of bifunctional degraders as elegant tools to alter the intracellular fate of proteins and to increase their MHC class I presentation in relevant disease models *in vitro* as well as *in vivo*.

## Author Contributions

SM designed and performed experiments, interpreted data, and wrote the manuscript; JV performed experiments and gave critical input; DB contributed the molecule dTAG-7 and critically revised the manuscript; GW contributed the molecule dTAG-7, provided strategical advice and critically revised the manuscript; CS initiated and supervised the study, designed and performed experiments, interpreted and revised data, and wrote the manuscript.

## Conflict of Interest Statement

DB is a current employee of Novartis Institutes of Biomedical Research. The other authors declare no conflict of interest.

## Supplementary Material

Figure S1 Uncropped western blot images used for Figure 1.

Figure S2 The effect of degradation tag (dTAG)-7 treatment on total MHC class I surface expression. **(A–C)** BMC-2 cells, either wildtype (WT, left side) or stably expressing GFP-S8L-F12 (right side), were treated for indicated time points with 1 µM dTAG-7 or 0.1% DMSO as control. Subsequently, GFP-specific fluorescence (MFI-GFP) **(A)**, as well as surface expression of the MHC class I molecules H-2K^b^ **(B)** and H-2D^b^ **(C)** was quantified using flow cytometry. This experiment was performed in triplicates and results are depicted as mean ± SD. Representative results from three independent experiments are shown.
