# Supplementary figures and images for: Acute Pharmacologic Degradation of a Stable Antigen Enhances Its Direct Presentation on MHC Class I Molecules
Source: Front Immunol. 2018 Jan 8;8:1920. doi: 10.3389/fimmu.2017.01920 (PMC5766668; doi:10.3389/fimmu.2017.01920)

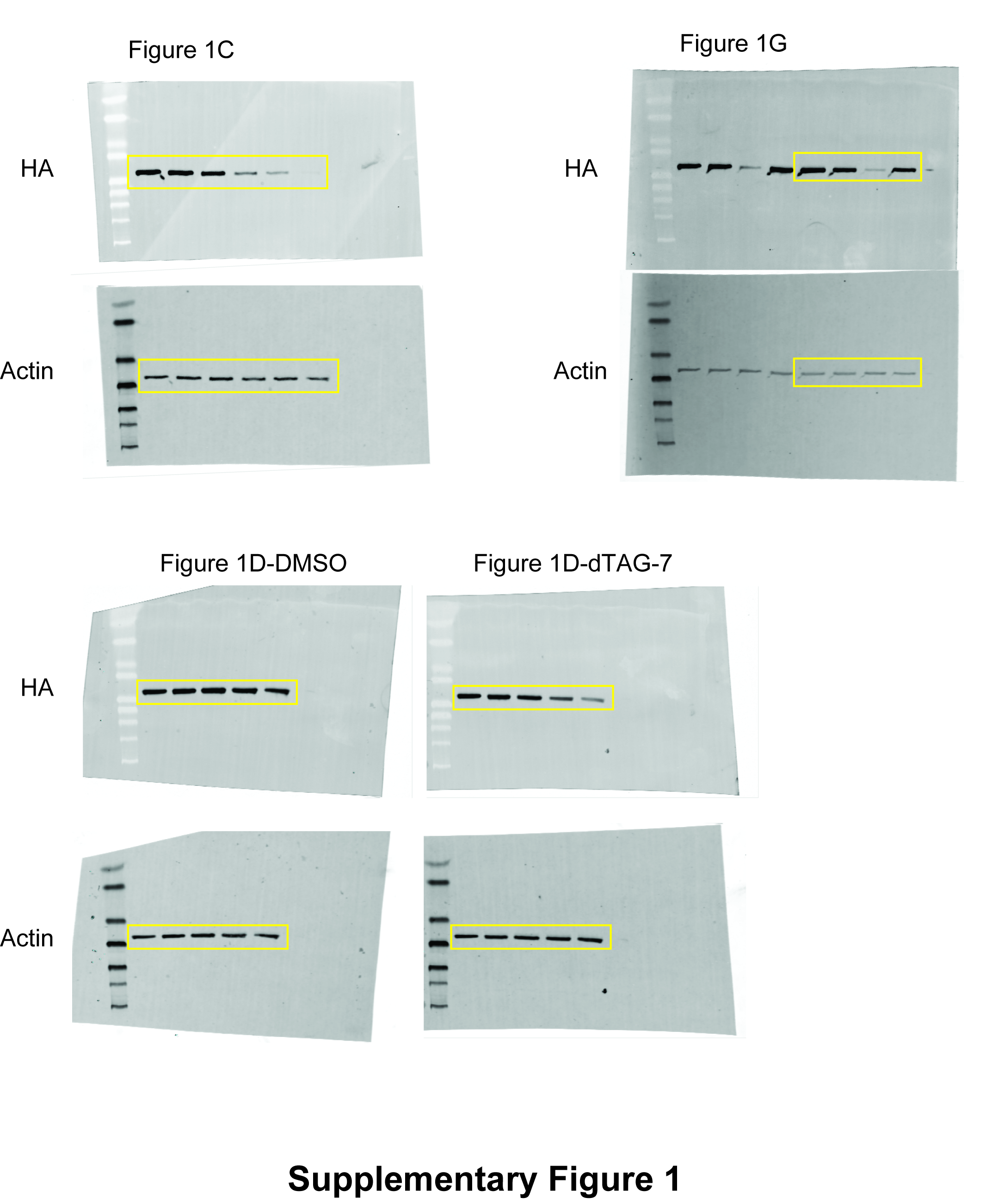

Supplement: Figure S1 — Uncropped western blot images used for Figure 1. [file Image_1.tif]

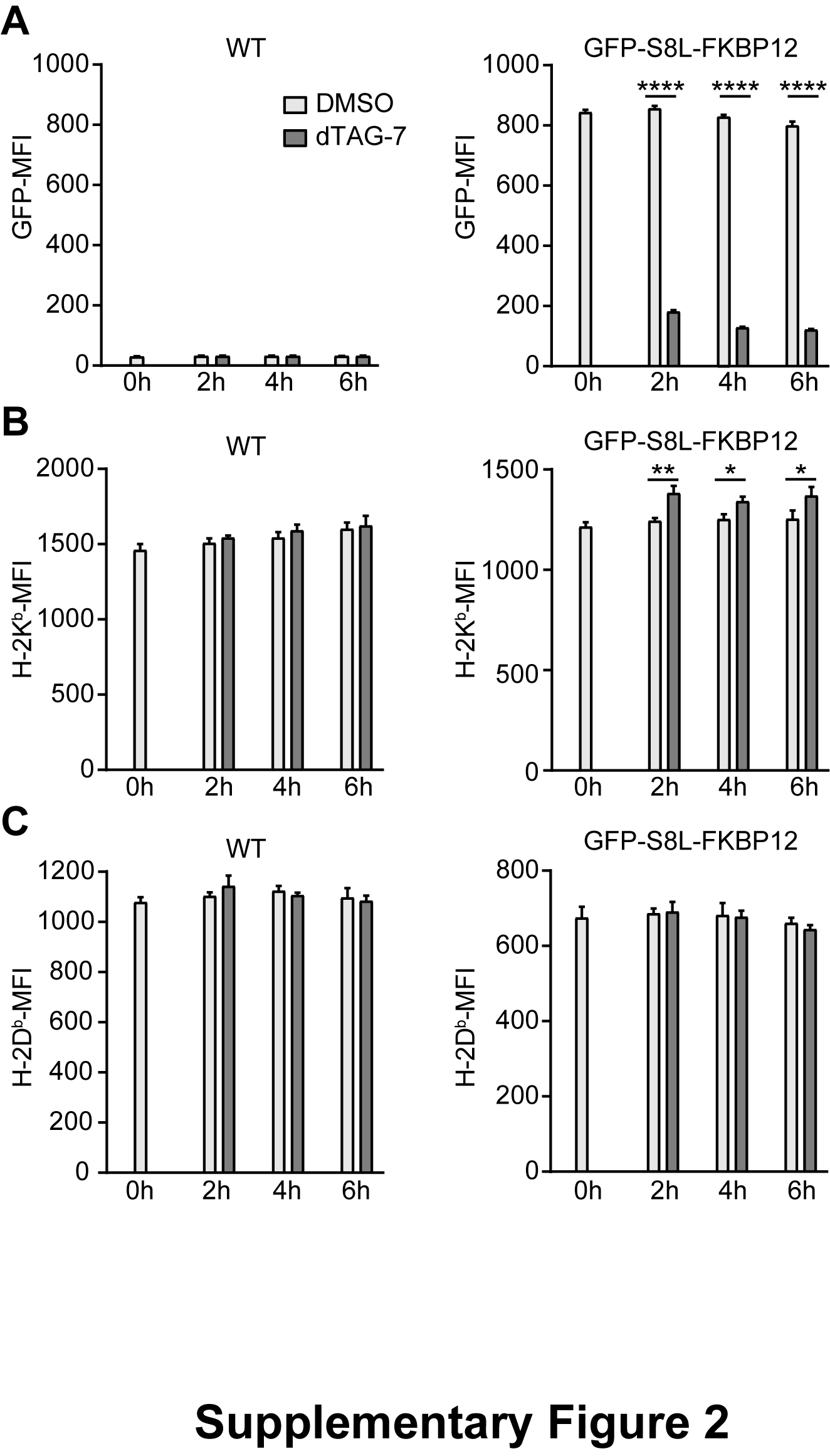

Supplement: Figure S2 — The effect of degradation tag (dTAG)-7 treatment on total MHC class I surface expression. (A–C) BMC-2 cells, either wildtype (WT, left side) or stably expressing GFP-S8L-F12 (right side), were treated for indicated time points with 1 µM dTAG-7 or 0.1% DMSO as control. Subsequently, GFP-specific fluorescence (MFI-GFP) (A), as well as surface expression of the MHC class I molecules H-2Kb (B) and H-2Db (C) was quantified using flow cytometry. This experiment was performed in triplicates and results are depicted as mean ± SD. Representative results from three independent experiments are shown. [file Image_2.tif]
